# Development and Internal Validation of Supervised Machine Learning Algorithms for Predicting the Risk of Surgical Site Infection Following Minimally Invasive Transforaminal Lumbar Interbody Fusion

**DOI:** 10.3389/fmed.2021.771608

**Published:** 2021-12-20

**Authors:** Haosheng Wang, Tingting Fan, Bo Yang, Qiang Lin, Wenle Li, Mingyu Yang

**Affiliations:** ^1^Department of Orthopedics, Taizhou Central Hospital (Affiliated Hospital to Taizhou College), Taizhou, China; ^2^Department of Orthopedics, Baoji City Hospital of Traditional Chinese Medicine, Baoji, China; ^3^Department of Endocrinology, Baoji City Hospital of Traditional Chinese Medicine, Baoji, China; ^4^Department of Orthopedics, Xianyang Central Hospital, Xianyang, China; ^5^Clinical Medical Research Center, Xianyang Central Hospital, Xianyang, China

**Keywords:** surgical infection sites, machine learning, risk factors, minimally invasive transforaminal lumbar interbody fusion, prediction model

## Abstract

**Purpose:** Machine Learning (ML) is rapidly growing in capability and is increasingly applied to model outcomes and complications in medicine. Surgical site infections (SSI) are a common post-operative complication in spinal surgery. This study aimed to develop and validate supervised ML algorithms for predicting the risk of SSI following minimally invasive transforaminal lumbar interbody fusion (MIS-TLIF).

**Methods:** This single-central retrospective study included a total of 705 cases between May 2012 and October 2019. Data of patients who underwent MIS-TLIF was extracted by the electronic medical record system. The patient's clinical characteristics, surgery-related parameters, and routine laboratory tests were collected. Stepwise logistic regression analyses were used to screen and identify potential predictors for SSI. Then, these factors were imported into six ML algorithms, including k-Nearest Neighbor (KNN), Decision Tree (DT), Support Vector Machine (SVM), Random Forest (RF), Multi-Layer Perceptron (MLP), and Naïve Bayes (NB), to develop a prediction model for predicting the risk of SSI following MIS-TLIF under Quadrant channel. During the training process, 10-fold cross-validation was used for validation. Indices like the area under the receiver operating characteristic (AUC), sensitivity, specificity, and accuracy (ACC) were reported to test the performance of ML models.

**Results:** Among the 705 patients, SSI occurred in 33 patients (4.68%). The stepwise logistic regression analyses showed that pre-operative glycated hemoglobin A1c (HbA1c), estimated blood loss (EBL), pre-operative albumin, body mass index (BMI), and age were potential predictors of SSI. In predicting SSI, six ML models posted an average AUC of 0.60–0.80 and an ACC of 0.80–0.95, with the NB model standing out, registering an average AUC and an ACC of 0.78 and 0.90. Then, the feature importance of the NB model was reported.

**Conclusions:** ML algorithms are impressive tools in clinical decision-making, which can achieve satisfactory prediction of SSI with the NB model performing the best. The NB model may help access the risk of SSI following MIS-TLIF and facilitate clinical decision-making. However, future external validation is needed.

## Introduction

Minimally invasive transforaminal lumbar interbody fusion (MIS-TLIF) is a classic minimally invasive operation for the treatment of lumbar degenerative diseases such as lumbar disc herniation, lumbar spinal stenosis, and lumbar spondylolisthesis. The operation uses channels to complete decompression and bone graft fusion and fixation. Compared with the traditional open TLIF, it better retains the paravertebral muscle structure, less intraoperative bleeding and faster post-operative recovery (1–3). It has become the mainstream operation scheme for minimally invasive fusion surgery at present.

Although surgical site infections (SSI) following MIS-TLIF are lower than those following open TLIF, previous literature has reported that the incidence of SSI after MIS-TLIF is not uncommon as this procedure is widely used in the clinic (4, 5). Surprisingly, over 20% of the patients who were re-admitted within 30 days after operation were caused by SSI (6, 7). Meanwhile, the rise in the number of spinal surgeries results in an expanding number of SSI, which poses a critical challenge to patients and clinicians. At present, there are studies on the analysis of pre-operative and intraoperative risk factors for SSI after total spinal open surgery, but there is little literature investigating the risk factors for SSI following MIS-TLIF. Previously, most studies only described these risk factors as relative risks (RR) or odds ratios (OR) (7, 8), which are not sufficient to evaluate the risk of SSI following MIS-TLIF. Therefore, a comprehensive prediction model combining risk factors is needed to assess SSI risk for patients. Identification of high-risk surgical populations might help target interventions to patients at high risk, reduce the risk of hospitalization, and improve the clinical outcome.

Machine learning (ML) is a form of artificial intelligence (AI) in which algorithms automatically learn and improve by identifying patterns and complex relationships, with the ultimate goal of making decisions using minimal human intervention (9–13). Through the good performance exhibited by ML algorithms in medical big data, we have the potential to obtain more superior prediction tools than traditional statistical modeling modalities under certain conditions for better prediction of the risk of SSI. It is frustrating that there are currently very few studies training ML algorithms to predict SSI risk. As such, this study aimed to develop and validate ML-based models using pre-operative and intraoperative variables to predict the risk of SSI following MIS-TLIF.

## Methods

### Patients

The study was approved by the institutional review board (IRB) of Taizhou Central Hospital and was performed following national and international guidelines. The ethics committee waived the requirement for informed consent due to the retrospective nature of the study. A total of 705 patients, referred from May 2012 to October 2019 to the Department of Orthopedics, Taizhou Central Hospital (Taizhou, China) for MIS-TLIF owing to lumbar spine diseases, were enrolled in this research. The selection criteria were as follows: (1) age ≥ 18 years; (2) diagnosis of lumbar degenerative disease, including lumbar disc herniation, lumbar spinal stenosis, lumbar spondylolisthesis, and lumbar instability; (3) undergoing primary single-or multi-segment MIS-TLIF. The exclusion criteria were as follows: (1) Patients were pre-operatively complicated by active infection in the spine or other parts of the body, cauda equina syndrome, spinal deformity, and tumors. (2) Emergency surgery.

All operations were done by 4 experienced spinal surgeons at our institution. The operation was performed using general anesthesia. C-arm X-ray fluoroscopy accurately located the lesion in the intervertebral space and the root of the upper and lower arches of the vertebrae. A longitudinal incision of ~2.0–3.0 cm of skin and deep fascia was made on the more symptomatic side of the patient along the previously marked pedicle line. The soft tissue between the longest muscle and the multifidus interval was bluntly separated until the vertebral plate was exposed. The Quadrant channel (Medtronic Inc. USA) was placed in the appropriate position after gradual expansion of the cannula, the free arm and cold light source were attached and secured to fully expose the articular eminence, the vertebral plate, and other important anatomic structures. The Quadrant channel was opened, the soft tissue was removed from the field of view under direct vision, and bipolar electrocoagulation was used to adequately stop the hemorrhage. The base diameter is enlarged depending on the reality and was generally dilated to 8–9 cm. The superior lamina and some articular processes were removed by nucleus pulposus forceps, the ligamentum flavum was cleaned, the dura was revealed, and the nerve roots were protected and freed. Further revealing the disc space, cutting through the annulus fibrosus, enucleating the nucleus pulposus, and using reamers and curettes to further clean the intervertebral space. A polyetheretherketone (PEEK) interbody graft (Capstone, Medtronic Sofamor Danek) was then inserted. Finally, with the help of the Sextant system (Medtronic Sofamor Danek), percutaneous lumbar pedicle screws were inserted bilaterally. A drainage tube was placed after washing, and then the layer was sutured and the incision was closed. The protocol for prevention of SSI in this central was described in more detail in Supplementary Material. Patients were followed for a minimum of 1 year after the index operation to monitor all complications and incidences of revision surgery. All complications were recorded by the available electronic medical system. The primary outcome of interest was the occurrence of SSI. The diagnosis of SSI was based on the CDC criteria (Center for Disease Control and Prevention) (14). The primary diagnostic criteria for SSI were as follows: (1) the surgical wound showed redness, swelling, heat, pain, wave sense or combined with abscess, and drooling, (2) Bacterial culture of wound exudate was positive, (3) Patients with re debridement surgery, positive intraoperative lavage fluid or tissue bacterial culture, and (4) Laboratory tests for blood routine, C-reactive protein, procalcitonin, as well as MRI examination or histopathological examination were performed to confirm SSI.

### Data Collection

The personal and medical information of patients, including age, body mass index (BMI), smoking, alcohol, chronic heart failure (CHF), diabetes, COPD, stroke, rheumatic disease, and diagnosis. The pre-operative laboratory examinations, including blood routine examination, glycated hemoglobin A1c (HbA1c), liver function test, serum potassium, and serum calcium, were collected from the electronic medical record (EMR) system. Meanwhile, we recorded the surgery-related parameters, including operation time, intraoperative blood loss, surgical level, and the number of fusion segments in this investigation.

### Statistical Analysis

The statistical analyses were performed using SPSS for Windows software (ver. 26.0; SPSS Inc., Chicago, IL, USA). For continuous, normally distributed variables, means were calculated and compared using the independent student *t*-test; otherwise, the Mann–Whitney *U*-test was used for group comparisons. The chi-square test was used to analyze qualitative variables. Values of *P* < 0.05 were considered significant.

### Training and Evaluation of ML Models

In this study, univariate and multivariate logistic regression tests were performed to identify the factors related to SSI following MIS-TLIF. Then, factors that were significant in both univariate and multivariate logistic regression were included and analyzed by stepwise regression. Next, the factors selected by stepwise regression are used as input variables to construct the machine learning (ML) models.

In its most basic sense, machine learning uses programmed algorithms that learn and optimize their operations by analyzing input data to make predictions within an acceptable range (15–18). In this study, 10-fold cross-validation was adopted, which means patients were randomly divided into a training set and a validation set at a ratio of 9:1 in each round. After 10 rounds, where all the patients are used for the test, the evaluation process ends. We developed five types of ML algorithms to model our data: k-Nearest Neighbor (KNN), Decision tree (DT), Support vector machine (SVM), Random Forest (RF), Multi-Layer Perceptron (MLP), Naïve Bayes (NB). When training the prediction model, we used 10-fold cross-validation (CV) on the training data to avoid overfitting and find the optimal hyperparameters. Then, Python software was used to further train the ML algorithms. To assess the performance of the ML algorithms, the area under the receiver operating characteristic (AUC) value, and sensitivity, specificity, and the whole dataset accuracy (ACC) of ML algorithms were reported. Of these, the ACC, sensitivity, and specificity were determined by the following formula:


$$\begin{array}{l} \;ACC=\frac{TP+TN}{TP+TN+FP+FN}\\ Sensitivity=\frac{TP}{TP+FN}\\ Specificity=\frac{TN}{FP+TN} \end{array}$$

**Figure 1:**
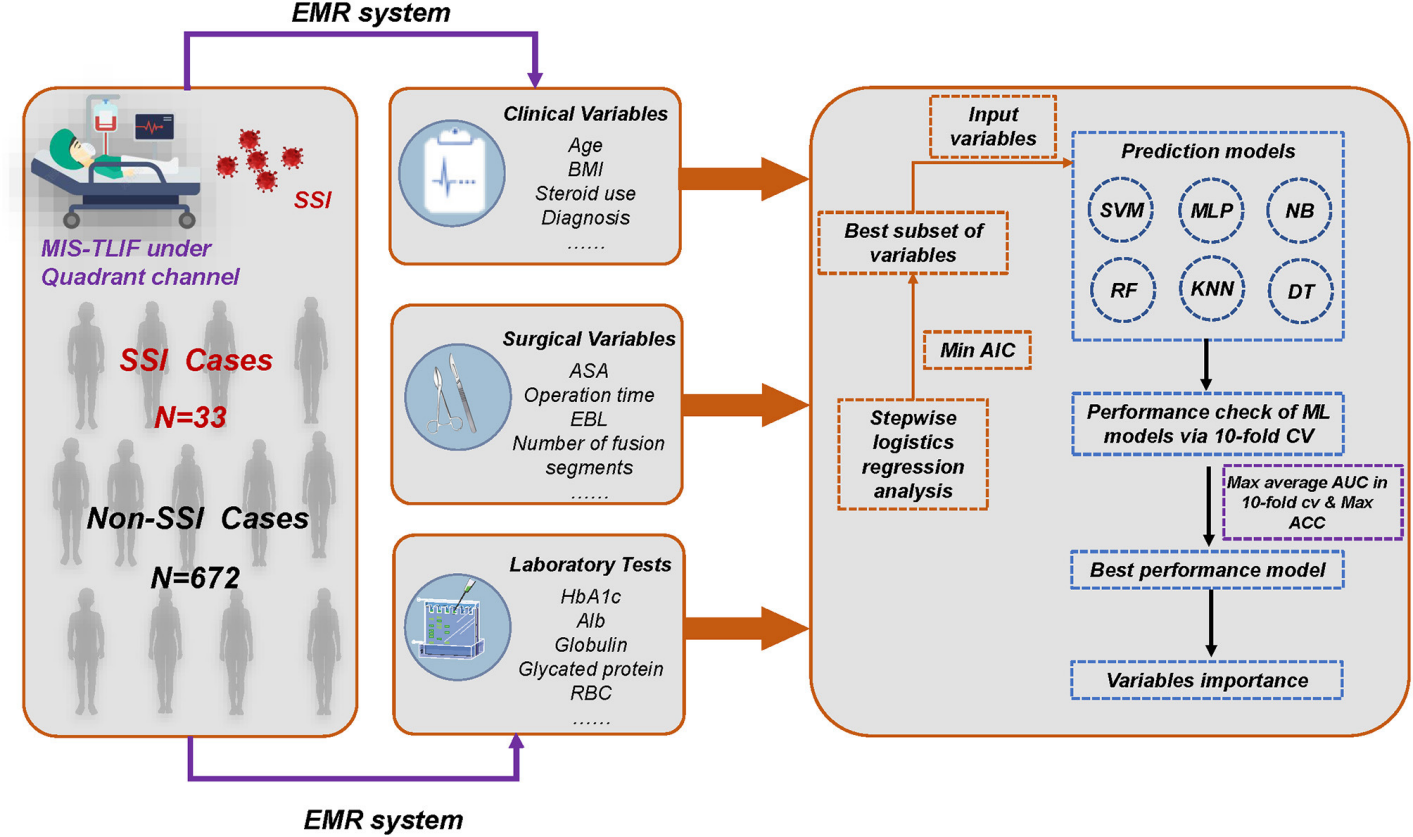
Overall flowchart of whole process. The final prediction model was determined by the result of maximum AUC and ACC. MIS-TLIF, minimally invasive transforaminal lumbar interbody fusion; SSI, surgical site infection; EMR, electronic medical record; AIC, Akaike information criterion; AUC, area under the curve; ACC, accuracy; KNN, k-Nearest Neighbor; DT, Decision tree; SVM, Support vector machine; RF, Random Forest; MLP, Multi-Layer Perceptron; NB, Naïve bayes.

In these formulas, TP: true positive; TN: true negative; FP: false positive; and FN: false negative. When we evaluated the performance of different ML algorithms, the closer the AUC was to 1, the better the classification performance of the model. In general, AUC > 0.7 indicated good performance of the model. We next determined the AUC values by comparing different ML algorithms, with the best performing algorithm as the final predictive model. Data analysis was performed using programs written in Python programming. The Python packages, including Scikit-learn 0.24.2 (19) and Python 3.8, are adopted for ML algorithms. A two-tailed *P* < 0.05 was deemed statistically significant. The flowchart of the study is presented in Figure 1.

## Results

### Patients' Characteristics

In this study, a total of 705 cases were included, 401 (56.9%) males and 304 (43.1%) females, mean (58.7 ± 7.2) years old. Among the 705 cases, SSI occurred in 33 (4.68%) cases, i.e., the SSI group; other cases were in the Non-SSI group. Among the SSI and Non-SSI groups, there were statistically significant differences in age (*p* < 0.001), BMI (*p* < 0.001), smoking (*p* = 0.002), diabetes (*p* < 0.001), ASA (*p* = 0.005), operation time (*p* < 0.001), estimated blood loss (EBL) (*p* < 0.001), number of fusion segments (*p* < 0.001), pre-operative HbA1c (*p* < 0.001), pre-operative albumin (*p* < 0.001), and pre-operative fibrinogen (*p* < 0.001). There were no significant differences in remaining parameters between the SSI and Non-SSI groups. The detailed results of the parameters are shown in Table 1.

**Table 1:** Comparison of variables between the SSI group and non-SSI group.

		**Total**	**Non-SSI**	**SSI**	***P-*value**
Number of patients		705	672	33	
Age (years)		58.7 (7.2)	58.4 (7.1)	65.0 (6.5)	<0.001^***^
**Sex (%)**					
	Female	304 (43.1)	289 (43.0)	15 (45.5)	0.858
	Male	401 (56.9)	383 (57.0)	18 (54.5)	
BMI (kg/m^2^)		25.2 (3.0)	25.0 (3.0)	28.4 (2.7)	<0.001^***^
**Smoking (%)**					
	No	519 (73.6)	503 (74.9)	16 (48.5)	0.002^**^
	Yes	186 (26.4)	169 (25.1)	17 (51.5)	
**Alcohol (%)**					
	No	479 (67.9)	457 (68.0)	22 (66.7)	0.851
	Yes	226 (32.1)	215 (32.0)	11 (33.3)	
**Diabetes (%)**					
	No	584 (82.8)	566 (84.2)	18 (54.5)	<0.001^***^
	Yes	121 (17.2)	106 (15.8)	15 (45.5)	
**COPD (%)**					
	No	680 (96.5)	648 (96.4)	32 (97.0)	1
	Yes	25 (3.5)	24 (3.6)	1 (3.0)	
**Stroke (%)**					
	No	697 (98.9)	664 (98.8)	33 (100.0)	1
	Yes	8 (1.1)	8 (1.2)	0 (0.0)	
**Rheumatic disease (%)**					
	No	687 (97.4)	655 (97.5)	32 (97.0)	0.583
	Yes	18 (2.6)	17 (2.5)	1 (3.0)	
**CHF (%)**					
	No	702 (99.6)	669 (99.6)	33 (100.0)	1
	Yes	3 (0.4)	3 (0.4)	0 (0.0)	
**Steroid use (%)**					
	No	646 (91.6)	615 (91.5)	31 (93.9)	1
	Yes	59 (8.4)	57 (8.5)	2 (6.1)	
**Diagnosis (%)**					
	Lumbar disc herniation	176 (25.0)	168 (25.0)	8 (24.2)	0.543
	Lumbar spinal stenosis	144 (20.4)	134 (19.9)	10 (30.3)	
	Lumbar instability/spondylolisthesis	169 (24.0)	162 (24.1)	7 (21.2)	
	Lumbar instability/spondylolisthesis combined with spinal stenosis	216 (30.6)	208 (31.0)	8 (24.2)	
ASA (%)	I	250 (35.5)	242 (36.0)	8 (24.2)	0.005^**^
	II	327 (46.4)	316 (47.0)	11 (33.3)	
	III	114 (16.2)	101 (15.0)	13 (39.4)	
	IV	14 (2.0)	13 (1.9)	1 (3.0)	
Operation time (min)		155.1 (26.7)	153.2 (24.5)	193.8 (37.4)	<0.001^***^
EBL (ml)		209.4 (63.3)	205.1 (58.4)	296.7 (91.9)	<0.001^***^
Number of fusion segments		1.1 (0.2)	1.1 (0.2)	1.4 (0.3)	<0.001^***^
Pre-operative HbA1c (%)		5.4 (1.0)	5.3 (0.8)	7.5 (1.6)	<0.001^***^
Pre-operative Alb (g/L)		42.8 (3.5)	43.0 (3.4)	39.3 (3.6)	<0.001^***^
Pre-operative globulin (g/L)		24.1 (21.9, 26.3)	24.1 (21.8, 26.2)	24.6 (22.2, 27.5)	0.17
Pre-operative glycated protein (%)		2.2 (0.1)	2.2 (0.1)	2.1 (0.2)	0.001^**^
Pre-operative RBC (^*^10^9^/L)		5.1 (4.8, 5.4)	5.1 (4.9, 5.4)	5.0 (4.8, 5.1)	0.079
Pre-operative WBC (^*^10^9^/L)		7.0 (6.1, 7.8)	7.0 (6.0, 7.8)	7.4 (6.7, 7.9)	0.07
Pre-operative thrombocyte (^*^10^9^/L)		238.7 (45.5)	238.7 (45.8)	238.3 (38.8)	0.965
Pre-operative PT (s)		11.6 (1.0)	11.6 (1.0)	11.8 (1.2)	0.249
Pre-operative APTT (s)		31.6 (1.3)	31.6 (1.2)	31.9 (2.0)	0.203
Pre-operative Fib (mg/dL)		3.6 (0.4)	3.6 (0.4)	3.2 (0.5)	<0.001^***^
Pre-operative potassium (mmol/L)		4.1 (0.3)	4.1 (0.3)	4.1 (0.3)	0.503
Pre-operative calcium (mmol/L)		2.4 (0.1)	2.4 (0.1)	2.4 (0.1)	0.504

*BMI, body mass index; COPD, chronic obstructive pulmoriary disease; CHF, chronic heart failure; ASA, American Society of Anesthesiologists; EBL, estimated blood loss; HbA1c, glycated hemoglobin A1c; Alb, albumin; RBC, red blood cells; WBC, white blood cells; PT, prothrombin time; APTT, activated partial thromboplastin time; Fib, fibrinogen*.

* *P-value < 0.05;*

** *P-value < 0.01;*

*** *P-value < 0.001*.

### Logistic Regression Analyses of SSI

In univariable analysis, age, BMI, smoking, diabetes, ASA, operation time, EBL, number of fusion segments, pre-operative HbA1c, pre-operative Alb, pre-operative glycated protein, and pre-operative fibrinogen were all significantly associated with the occurrence of SSI in the overall population (*P* < 0.05; Table 2). In multivariable logistic regression analysis, factors with statistical significance included age, BMI, operation time, EBL, pre-operative HbA1c, and pre-operative Alb (*P* < 0.05; Table 2). Stepwise logistic regression analyses identified the optimal subset of variables including age (OR 1.187, 95% CI 1.006–1.401), BMI (OR 1.445, 95% CI 1.005–2.076), pre-operative HbA1c (OR 35.390, 95% CI 21.215–40.124), EBL (OR 1.424, 95% CI 1.021–1.732), operation time (OR 1.041, 95% CI 1.003–1.080) (Table 3).

**Table 2:** Univariate and multivariate logistic regression model analyses of SSI following MIS-TLIF in the whole data set.

	**Univariable logistic regression analysis**		**Multivariable logistic regression analysis**	
	**OR (95% CI)**	***P*-value**	**OR (95% CI)**	***P-*value**
Age (years)	1.145 (1.085–1.207)	<0.001^***^	1.213 (1.091–1.334)	0.034^*^
Sex (%)	0.905 (0.449–1.827)	0.782	**/**	**/**
BMI (kg/m^2^)	1.498 (1.303–1.722)	<0.001^***^	1.521 (1.091–1.944)	0.0121^*^
Smoking (%)	3.162 (1.563–6.397)	0.001^***^	2.965 (2.504–3.426)	0.0654
Alcohol (%)	1.063 (0.506–2.231)	0.872	**/**	**/**
Diabetes (%)	2.450 (2.175–9.104)	<0.001^***^	3.054 (1.498–4.160)	0.0598
COPD (%)	0.844 (0.111–6.431)	0.87	**/**	**/**
Stroke (%)	0.000 (0.000- Inf)	0.987	**/**	**/**
Rheumatic disease (%)	1.204 (0.155–9.332)	0.859	**/**	**/**
CHF (%)	0.000 (0.000- Inf)	0.988	**/**	**/**
Steroid use (%)	0.696 (0.162–2.984)	0.626	**/**	**/**
Diagnosis (%)	0.892 (0.661–1.204)	0.454	**/**	**/**
ASA (%)	1.849 (1.201–2.847)	0.005^**^	1.456 (1.062–1.849)	0.1652
Operation time (min)	1.055 (1.040–1.071)	<0.001^***^	1.061 (1.032–1.090)	0.0254^*^
EBL (ml)	1.822 (1.015–2.785)	<0.001^***^	1.709 (1.368–2.050)	0.0197^*^
Number of fusion segments	2.292 (1.080–4.863)	0.031^*^	2.715 (2.017–3.413)	0.5681
Pre-operative HbA1c (%)	11.394 (5.876–22.092)	<0.001^***^	36.125 (27.899–44.351)	<0.001^***^
Pre-operative Alb (g/L)	0.747 (0.673–0.830)	<0.001^***^	0.621 (0.456–0.786)	0.0198^*^
Pre-operative globulin (g/L)	1.079 (0.969–1.202)	0.167	**/**	**/**
Pre-operative glycated protein (%)	0.010 (0.001–0.168)	0.001^**^	0.042 (0.026–0.058)	0.319
Pre-operative RBC (*10^9^/L)	0.526 (0.220–1.258)	0.149	**/**	**/**
Pre-operative WBC (*10^9^/L)	1.250 (0.945–1.655)	0.118	**/**	**/**
Pre-operative thrombocyte (*10^9^/L)	1.000 (0.992–1.008)	0.965	**/**	**/**
Pre-operative PT (s)	1.221 (0.870–1.714)	0.249	**/**	**/**
Pre-operative APTT (s)	1.195 (0.909–1.573)	0.203	**/**	**/**
Pre-operative Fib (mg/dL)	0.074 (0.029–0.190)	<0.001^***^	0.096 (0.041–0.151)	0.429
Pre-operative potassium (mmol/L)	0.685 (0.226–2.071)	0.502	**/**	**/**
Pre-operative calcium (mmol/L)	0.358 (0.018–7.247)	0.503	**/**	**/**

*BMI, body mass index; COPD, chronic obstructive pulmoriary disease; CHF, chronic heart failure; ASA, American Society of Anesthesiologists; EBL, estimated blood loss; HbA1c, glycated hemoglobin A1c; Alb, albumin; RBC, red blood cells; WBC, white blood cells; PT, prothrombin time; APTT, activated partial thromboplastin time; Fib, fibrinogen; OR, odds ratio; CI, confidence interval*.

* *P-value < 0.05;*

** *P-value < 0.01;*

*** *P-value < 0.001*.

**Table 3:** Stepwise logistics regression analysis in the whole data set.

	**Estimate**	**Std. error**	**OR**	**95% CI lower**	**95% CI upper**	***P*-value**
(Intercept)	−8.165	0.654	19.321	11.410	27.232	0.080
Age (years)	0.116	0.064	1.187	1.006	1.401	0.043^*^
BMI (kg/m^2^)	0.387	0.140	1.445	1.005	2.076	0.047^*^
Operation time (min)	0.032	0.013	1.041	1.003	1.080	0.035^*^
EBL (ml)	0.026	0.007	1.424	1.021	1.732	0.017^*^
Pre-operative HbA1c (%)	2.242	0.466	35.390	21.215	40.124	<0.001^***^
Pre-operative Alb (g/L)	−0.442	0.133	0.672	0.463	0.975	0.036^*^

*BMI, body mass index; EBL, estimated blood loss; HbA1c, glycated hemoglobin A1c; Alb, albumin; OR, odds ratio; CI, confidence interval*.

* *P-value < 0.05;*

*** *P-value <0.001*.

### Predictive ML Algorithms Performance

**Figure 2:**
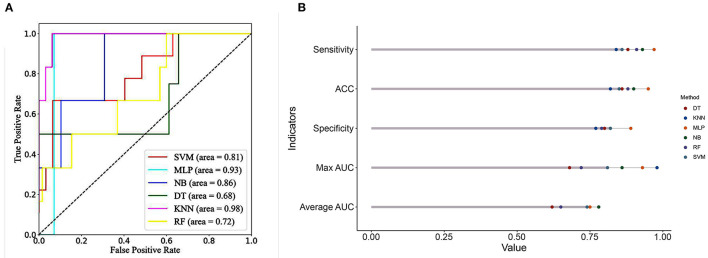
**(A)** ROC curve analysis of machine learning algorithms for predicting the risk of SSI following MIS-TLIF in total set. **(B)** Predictive performance comparison of the six types of machine learning algorithms in the total set. SSI, surgical site infection; MIS-TLIF, minimally invasive transforaminal lumbar interbody fusion; ROC, Receiver Operating Characteristic curve; KNN, k-Nearest Neighbor; DT, Decision tree; SVM, Support vector machine; RF, Random Forest; MLP, Multi-Layer Perceptron; NB, Naïve bayes.

**Figure 3:**
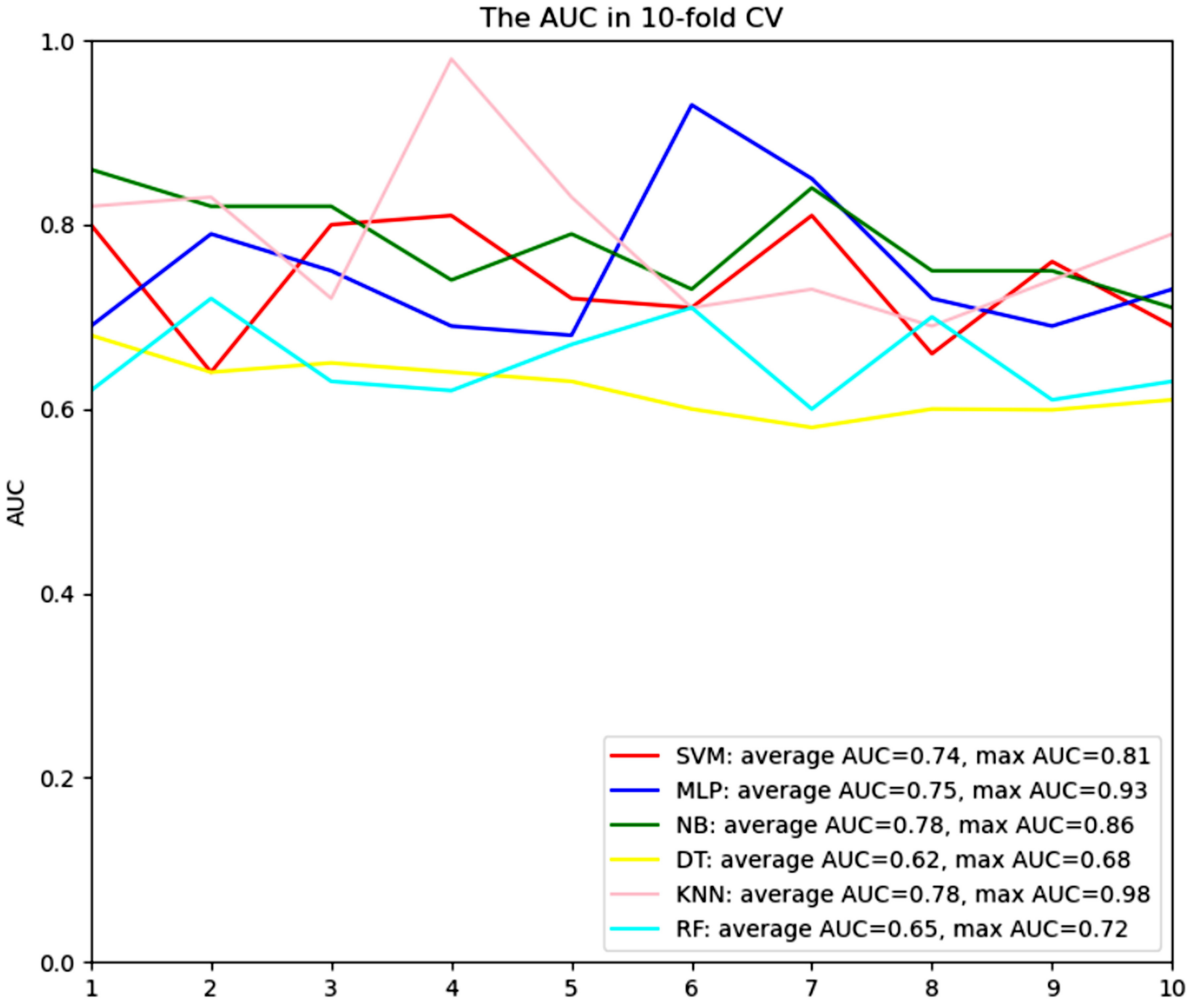
ROC curve analysis of a 10-fold CV of six machine learning algorithms for predicting the risk of surgical site infection following minimally invasive transforaminal lumbar interbody fusion in total set. CV, cross validation; KNN, k-Nearest Neighbor; DT, Decision tree; SVM, Support vector machine; RF, Random Forest; MLP, Multi-Layer Perceptron; NB, Naïve bayes.

Comparisons of the performance of prediction among the six ML algorithms models in the whole set are detailed in Figure 2. The result of the 10-fold CV of six ML algorithms in the whole set was demonstrated in Figure 3. It turned out that the NB model demonstrated the highest performance in predicting SSI, whose average AUC was 0.78, sensitivity 0.93, specificity 0.82, and accuracy 0.90 in whole sets. Accordingly, we chose the NB model as the final prediction model.

### Relative Importance of Variables

**Figure 4:**
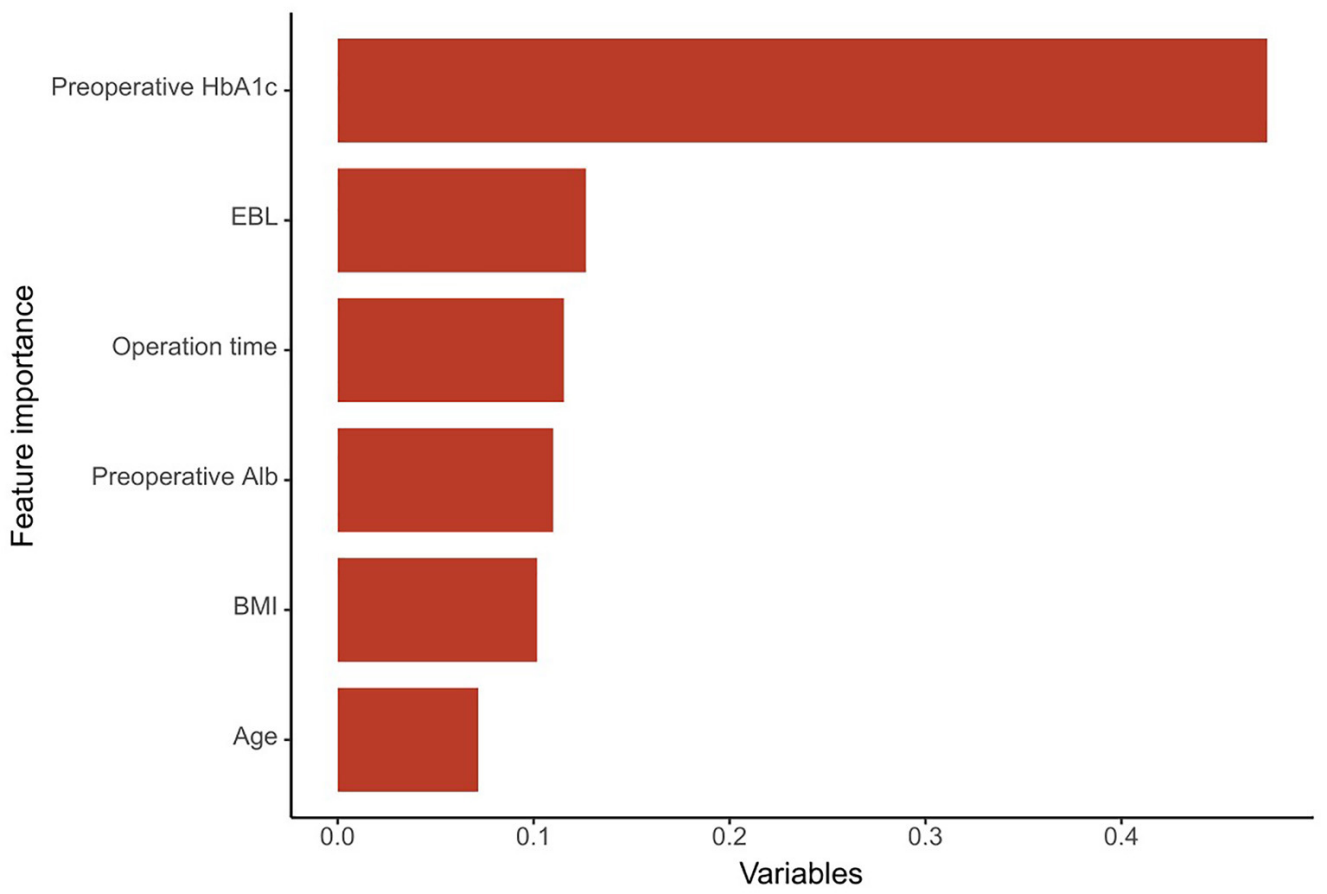
The importance of the variables in the NB model model is in decreasing order as follows: Pre-operative HbA1c, EBL, operation time, pre-operative Alb, BMI, and age. HbA1c, glycated hemoglobin A1c; EBL, estimated blood loss; Alb, albumin; BMI, body mass index.

According to the indicators, the NB model is determined as the final prediction model. The relative importance of variables in the NB model was shown in Figure 4. The importance of high-ranking variables in the NB model was arranged as follows in descending order: pre-operative HbA1c, EBL, operation time, pre-operative Alb, BMI, and age.

## Discussion

In this work, we developed and validated multiple ML algorithms to predict the risk of SSI following MIS-TLIF under the Quadrant channel. We found that the NB model provided improved predictive ability compared to other models. This study produced interesting findings concerning the added value of machine-learning methods, contrary to some of the wider literature on clinical prediction models. This ML-based model may potentially assist clinical decision-making when assessing and advising patients after MIS-TLIF under the Quadrant channel and when deciding on ongoing management.

MIS-TLIF is well-known to have many advantages compared to open TLIF in terms of reducing muscle injury, smaller incisions, and scarring, especially for elderly patients who cannot bear the trauma of traditional open TLIF surgery (20–22). In contrast, SSI after MIS-TLIF can cause additional prolonged antibiotic use, prolonged length of stay, increased risk of fusion failure, and even the need for re-invasive surgery or removal of the implant (23, 24), which not only increases the medical burden on patients and society but also causes increased mortality in patients.

With the continuous development of minimally invasive spine surgery, MIS-TLIF has gradually attracted the attention of spine surgeons. It has been reported that the incidence of SSI after lumbar interbody fusion is 2.4–5% (25–28). In this investigation, the incidence of SSI was 4.78, which was consistent with the previous report. Although risk factors associated with SSI after MIS-TLIF have been previously reported, little attention has been paid to the incidence of SSI following MIS-TLIF under the Quadrant channel. More importantly, we still lack a concise and reliable tool to identify high-risk groups and assist clinicians in their decision-making. Therefore, in this study, several ML-based models were constructed using AI techniques to predict the risk of SSI.

Many studies have shown that diabetes mellitus is an important risk factor for SSI after spine surgery (23, 29–31). Usually, glucose metabolism is closely related to the structure and pathophysiology of blood vessels. Disorders of glucose metabolism are inextricably linked to vascular structure and dysfunction, as has been demonstrated in many studies. And vascular pathological alterations cause local microcirculatory disorders leading to tissue ischemia and hypoxia, which provide a favorable environment for the growth of anaerobic bacteria and pose a potential risk for delayed post-operative wound healing and surgical site infection. Also, systemic immune deficiency caused by diabetes is the main reason for the occurrence of SSI. Therefore, surgeons usually strictly control patients' perioperative blood glucose and control pre-operative fasting glucose below 6.9 mmol/L before considering surgical treatment (32). Interestingly, glycosylated hemoglobin has also been identified as a risk factor for SSI. Chronic hyperglycemic state leads to cell-mediated immune and macrophage dysfunction, reduced overall immune function of the body, and diminished ability to respond to bacterial viruses. And the relative importance in our prediction model was ranked first. Therefore, we need to be more cautious about the perioperative glycemic management of patients.

In the present study, we found that high BMI is also a risk factor for SSI. In a large retrospective study by Onyekwelu et al. (33), BMI > 30 kg/m^2^ was found to be a high-risk factor for SSI after lumbar spinal fusion. The problems caused by obesity are present in almost all orthopedic surgeries, not only in spine surgery. Obesity can lead to endocrine disruption and insulin resistance, causing impaired glucose tolerance and even diabetes. On the other hand, obese patients may have a variety of underlying diseases that cause a decrease in the patient's immune capacity. Equally worrisome is the increased vascularity in the adipose tissue of obese patients and the even greater incidence of post-operative fat liquefaction, which also increases the risk of SSI.

Intriguingly, age was also identified as an independent risk factor in this study. However, we are also aware that this is a non-modifiable risk factor. Previous studies reported that the risk of SSI after spinal surgery was three times higher in patients aged ≥ 60 years than in the younger group (1, 27). Similar results were observed in the present study. Although it is still controversial whether advanced age is a risk factor for SSI, the current mainstream of scholars believe that elderly patients have reduced metabolism, degenerated immune function, and may be accompanied by numerous underlying diseases, which significantly reduce the reserve capacity of the organism and make it more sensitive to surgical trauma strikes. The ability of local tissues to resist infection as well as self-repair is weakened, increasing the risk of SSI following spine surgery.

Although univariate logistics regression analysis indicated a statistical difference in operation time and EBL between the two groups (*P* < 0.05), in multivariate logistic regression analysis, we found that EBL and operation time were identified as risk factors for SSI. It has been reported in the literature that prolonged operation time is a significant risk factor for SSI that cannot be ignored. Prolonged operation time leads to increased tissue stretching time, aggravating local ischemia and tissue inflammatory factor release, while prolonged surgical wound exposure leads to an increased possibility of direct blood entry of pathogenic bacteria from the wound. Whereas, the relationship between EBL and SSI has been debatable. According to previous literature, intraoperative blood transfusion >1,000 ml was reported as a risk factor for post-operative SSI in spine surgery and cardiac surgery (7, 27). Onyekwelu et al. (33) investigated 3,175 patients undergoing spine surgery and found that the incidence of post-operative SSI was 2.5% in patients with intraoperative bleeding <1,000 ml, and 3.5% in patients with intraoperative bleeding >1,000 ml. The incidence of SSI was 5.6% (*p* = 0.001) and was identified as an independent risk factor for SSI.

In the present study, we found that low serum albumin was an important risk factor for SSI. Serum albumin level is closely related to the nutritional status, liver and kidney function of patients. Hypoproteinemia will lead to immune suppression, local tissue edema, impaired microcirculation, and increased tissue fluid exudation, which will affect normal wound healing and create conditions for bacterial colonization. Previous literature has reported pre-operative hypocalcemia as a risk factor for post-operative spinal infection (30), and another interesting finding is that electrolyte variables such as blood calcium and potassium in this investigation were not statistically significantly different between the SSI and Non-SSI groups. Patients with toxic shock syndrome are usually found to suffer from hypocalcemia, which may be due to an increase in calcitonin secretion caused by the infection, resulting in lower blood calcium levels (34). Many previous studies have reported hypocalcemia in ~40% of patients with bacteremia (35). According to the inclusion and exclusion criteria of our study, none of the patients in the 2 groups had a significant active infection before surgery, so it is not difficult to explain why there was no statistically significant difference in pre-operative blood calcium between the 2 groups.

Compared with other research attempting to determine the risk of SSI following MIS-TLIF, our work has several advantages. First, a few studies focused on patients undergoing MIS-TLIF under the Quadrant channel. This may provide a reference for spine surgeons. Furthermore, while ML approaches have shown unparalleled prediction performance in MIS-TLIF under the Quadrant channel in recent reports, There is, however, little research in the available literature on applying ML algorithms to SSI following MIS-TLIF. As far as we know, this is the first study to develop a prediction model using ML algorithms for risk evaluation of SSI with easy-to-use clinical and laboratory data. Fortunately, our prediction model evidences good predictive performance of the model, which distinguishes itself from linear models adopted by previous research.

In this study, several limitations of this study require attention. First, the nature of a retrospective study might have introduced a selection bias. Second, the ML algorithm model we established, to some extent, was confined to one single center, which might restrict its generalizability pending further validation in real-world scenarios. Third, further independent external validation is required to confirm these findings. Finally, we collected as comprehensive a collection of SSI-related variables as possible, but there were still some important variables, such as methicillin-resistant *Staphylococcus aureus* (MRSA) carriage, that were not available in a timely manner. This could limit the generalizability of the study. Future research was needed to further look into this issue.

## Conclusions

We developed and validated ML algorithms for individualized prediction of SSI following MIS-TLIF by utilizing readily available pre-operative variables. The ML-based prediction model can identify whether patients are at high risk of SSI and may assist the clinician in decision making. In the future, we hope to enlarge the sample size in the following study and obtain more reliable achievements further.

## Data Availability Statement

The original contributions presented in the study are included in the article/Supplementary Material, further inquiries can be directed to the corresponding author/s.

## Ethics Statement

The study was approved by an Institutional Ethics Committee at the Taizhou Central Hospital (Affiliated Hospital to Taizhou College). Considering that this work was a retrospective study: the Ethics Committee waived the requirement for informed consent from patients.

## Author Contributions

HW collected the data, analyzed the data, and drafted the manuscript. MY supervised the project, reviewed the manuscript, responsible for the whole project, designed the study, and supervised the study. BY, WL, TF, and QL conceived of the study, participated in its design and coordination, and helped to draft the manuscript. All authors read and approved the final manuscript.

## Conflict of Interest

The authors declare that the research was conducted in the absence of any commercial or financial relationships that could be construed as a potential conflict of interest.

## Publisher's Note

All claims expressed in this article are solely those of the authors and do not necessarily represent those of their affiliated organizations, or those of the publisher, the editors and the reviewers. Any product that may be evaluated in this article, or claim that may be made by its manufacturer, is not guaranteed or endorsed by the publisher.

## Abbreviations

BMI: body mass index
COPD: chronic obstructive pulmoriary disease
CHF: chronic heart failure
ASA: American Society of Anesthesiologists
EBL: estimated blood loss
HbA1c: glycated hemoglobin A1c
Alb: albumin
RBC: red blood cells
WBC: white blood cells
PT: prothrombin time
APTT: activated partial thromboplastin time
Fib: fibrinogen
MIS-TLIF: minimally invasive transforaminal lumbar interbody fusion
CV: cross-validation
KNN: k-Nearest Neighbor
DT: Decision tree
SVM: Support vector machine
RF: Random Forest
MLP: Multi-Layer Perceptron
NB: Naïve Bayes
ACC: accuracy
AUC: area under the receiver operating characteristic curve
CV: cross-validation
TP: true positive
TN: true negative
FP: false positive
FN: false negative
OR: odds ratio
CI: confidence interval
ML: machine learning
AI: artificial intelligence
EMR: electronic medical record
MRSA: methicillin-resistant *Staphylococcus aureus*.
