# Supplementary material for: Development and Internal Validation of Supervised Machine Learning Algorithms for Predicting the Risk of Surgical Site Infection Following Minimally Invasive Transforaminal Lumbar Interbody Fusion
Source: Front Med (Lausanne). 2021 Dec 20;8:771608. doi: 10.3389/fmed.2021.771608 (PMC8720930; doi:10.3389/fmed.2021.771608)
Supplement: Supplementary file 1 [file Presentation_1.ZIP › Supplementary Materials.docx]

**Protocol for Prevention of SSI in Orthopaedic Surgery**

----------------------------------------------------------------------------------------------------------------------**Preoperative Management**

----------------------------------------------------------------------------------------------------------------------

**1. Rational uses of antibiotics**

It has long been established that the appropriate use of prophylactic antibiotics can significantly reduce the incidence of SSI, and most guidelines available focus on treatment against gram-positive bacteria (staphylococci), with the antibiotic of choice being cefazolin, a first-generation cephalosporin with a long half-life in bone and blood that is mainly effective against gram-positive and some gram-negative bacteria. Nunez- Pereira S showed that the use of prophylactic antibiotics 30 min before surgery significantly reduced the risk of SSI. In addition, a study by Swoboda SM et al. showed that antibiotics need to be added every 4 hours if the duration of surgery is too long, and in procedures such as spinal orthopedics, where blood loss exceeds 1500 ml, an additional antibiotic is likewise required.

**2. Preoperative showering and bathing with chlorhexidine**

Prior to surgery, patients should shower at least 3 times with chlorhexidine. Several pre-surgical showers will prevent the growth of bacteria and provide the best protection when taken at least 3 times in a row. 3 showers are taken: the evening two days before surgery, the evening the day before surgery and the morning of the day of surgery.

**3. Tight glycemic control in individuals with diabetes**

Patients with diabetes mellitus should have strict glycemic control, especially for patients undergoing elective surgery, who can be instructed to control their blood glucose prior to admission so that their glycated hemoglobin will be below 7% before surgery.

**4. Smoking cessation**

To effectively reduce the incidence of postoperative SSIs, smoking cessation must begin 4 to 8 weeks before surgery. Quitting smoking for less than 4 weeks is not effective in reducing the risk of SSI.

**5. Reduce body weight**

In a 2013 Mata analysis, Abdallah et al. reported that obesity was identified as a risk factor for SSI, and a 5-point increase in body mass index (BMI) was associated with a 21% increased risk of postoperative spinal infection. Therefore, weight intervention and preoperative weight optimization for patients requiring elective spine surgery is essential to reduce SSI.

**6. Correction of hypoalbuminemia**

An albumin level below 3.5 g / dL is defined as hypoalbuminemia. Albumin levels allow the clinician to know the nutritional status of the patient preoperatively. Previously, the literature reported that albumin levels were an independent risk factor for delayed wound healing and for readmission. A retrospective analysis of 4310 patients by Bohl et al. showed that hypoalbuminemia was associated with postoperative infection and other complications of the wound in patients undergoing lumbar spinal fusion. Interestingly, hypoalbuminemia may also be present in obese patients. The exact mechanism of hypoproteinemia in obese patients is still controversial, and some scholars believe it is related to the inflammatory response, which leads to lower albumin levels. Therefore preoperative examination of albumin levels is necessary and needs to be corrected if it is below 3.5 g / dL.

---------------------------------------------------------------------------------------------------------------------- **Intraoperative Management**

----------------------------------------------------------------------------------------------------------------------

**1. Disinfection of hands**

Hand disinfection plays a crucial role in the prevention of SSI. There are two main types of hand disinfectants in common use today, chlorhexidine or povidone-iodine disinfectants.A study by Fletcher et al. summarized the latest evidence regarding hand disinfection for physicians in surgery and found that chlorhexidine-based disinfectant ointments were more effective in reducing the number of skin colonies, but did not make a significant difference in the incidence of postoperative SSI. The results of the Cochrane study noted that there was no conclusive evidence that any one hand disinfectant was significantly better than others in reducing SSI, but there was a significantly lower risk compared to the control group (i.e., no hand disinfector).

**2. Adequate skin disinfection of the surgical area**

The purpose of skin disinfection in the operative area is to sterilize the skin in the operative area prior to incision and to minimize the risk of bacterial contamination of the incision in the normal skin flora. A Mata analysis by Sidhwa F concluded that alcohol-based disinfectants may be superior to iodine and chlorhexidine, and that chlorhexidine reduces the incidence of postoperative SSI more than iodine, but the authors noted that the available evidence is of moderate quality and that further large prospective randomized controlled trials are needed to Further large prospective randomized controlled trials are needed to draw more definitive conclusions.

**3. Disposable double gloves**

The use of intraoperative surgical gloves protects the surgeon and reduces the risk of SSI. In a paper by Tanner J et al, it was concluded that double gloves significantly reduced the risk of glove perforation and that different colored gloves could help us to detect glove rupture earlier. The SSI was significantly reduced by wearing the same pair of gloves throughout the procedure.

**4. Intraoperative irrigation**

Masahiko W et al. divided into 3 groups (<1000 ml, 1000-2000 ml and >2000 ml) according to the average saline flush volume per hour and found that the incidence of postoperative SSI was significantly reduced when the average saline flush volume per hour was >2000 ml, so they recommended intraoperative flushing with at least 500 ml every 15 minutes They therefore recommended intraoperative saline flushing of at least 500 ml of saline every 15 minutes to prevent the development of postoperative SSI in spinal surgery. Several studies have confirmed the effectiveness of adding povidone to the irrigating saline to prevent the occurrence of postoperative SSI, but there is no consensus on how to use povidone to irrigate the surgical area. In a study by Onishi Y et al, it was recommended that the incision be irrigated with a 1% povidone/saline solution for about 90 seconds every 1.5 hours, followed by saline irrigation, and it was finally concluded that this method, compared with saline irrigation alone This method significantly reduces the risk of postoperative deep tissue SSI compared to saline flushing alone. In contrast, the study by Justin V.C. Lemans et al. in which a 1.3 g/L povidone iodine/saline solution was used at the end of the procedure showed no significant difference in the prevention of postoperative deep tissue SSI, but a significant difference in the prevention of superficial tissue SSI.

**5. Maintaining the patient's intraoperative body temperature**

Many previous studies have shown that intraoperative hypothermia is associated with an increased risk of SSI, possibly because hypothermia leads to reduced tissue perfusion, which leads to tissue hypoxia and increased susceptibility to infection. a study by Madrid E et al. showed that intraoperative administration of heat to patients had a significant effect on reducing the incidence of SSI and complications.

**6. Use of disposable surgical instruments**

In a study by Litrico S et al, single-use instrumentation was found to result in a significant reduction in the incidence of SSI after lumbar fusion. We believe that a single-use instrumentation solution is relatively feasible and economical. It keeps the implantable screws and rods in a sterile package until they are placed in the patient's pedicle. This greatly reduces the risk of their contamination by airborne bacteria in the operating room, whereas the standard instruments we often use for sterilization are exposed to the sterile table throughout the procedure, which increases the chance of contamination of the instruments by airborne bacteria. Therefore, if practical, instruments or internal fixations that are not used during surgery should be wrapped or covered with disposable sterile sheets whenever possible to reduce their chance of contamination by airborne bacteria.

**7. Triclosan-coated sutures**

Triclosan-coated polyglycolic lactic acid sutures are beneficial in limiting bacterial colonization and further reducing the incidence of SSI. In a retrospective analysis conducted by Ueno et al. comparing the incidence of SSI after surgery with conventional sutures and triclosan-coated sutures, it was shown that the incidence of SSI after surgery with conventional sutures was 3.90%, whereas the incidence of SSI after surgery with triclosan-coated sutures was 0.50% (P=0.02). The incidence of SSI was 0.50% with conventional sutures (P = 0.02), and it was concluded that triclosan-coated sutures significantly reduced the incidence of SSI after spinal surgery.

---------------------------------------------------------------------------------------------------------------------- **Postoperative Management**

----------------------------------------------------------------------------------------------------------------------

**1. Maintenance of satisfactory blood oxygen saturation**

It is well documented that postoperative administration of oxygen to patients to maintain good oxygen saturation is essential to reduce the incidence of SSI. in a Mata analysis done by Qadan M et al, data from 5 RCTs in 3001 patients were analyzed and it was concluded that maintaining a patient's postoperative oxygen saturation >80% reduced the RR (relative risk) of postoperative SSI by 25%. Maintaining good oxygen saturation maintains good immune system function in the body.

**2. Glucose management for diabetic patients**

For reducing the incidence of postoperative SSI, strict control of patient blood glucose levels preoperatively is necessary, and postoperative glycemic management of diabetic patients is equally important. Excessive blood glucose results in immunoglobulin glycosylation, which leads to decreased neutrophil function and reduced cellular bactericidal capacity. lipshutz and Glopper reviewed the analysis of perioperative glycemic management and they recommended postoperative control of glycemic levels below 150 mg/dL (approximately 8.3 mmol/L) in diabetic patients without the need to control patients' blood glucose to normal levels, and 150 mg/dL (approximately 6.1 mmol/L), although the latter may better prevent the occurrence of postoperative SSI relative to the former, but also of increasing the chance of adverse events such as hypoglycemia.

**3. Postoperative prophylactic antibiotics**

Although there is still more controversy, most scholars agree that prophylactic antibiotics can be used until 72h postoperatively for patients at high risk of SSI (e.g., diabetic patients, patients requiring internal fixation implants, etc.), with cefazolin as the antibiotic of choice.

**4. Postoperative drainage tube care**

First of all, for the prevention of SSI, whether the use of drains increases the incidence of SSI, several high-quality evidence-based publications have confirmed that the use of drains during spinal surgery does not increase the incidence of SSI. As for the duration of drain retention, a recent study by Zach Pennington et al. showed a significant reduction in the incidence of SSI after drain removal at 3 days postoperatively compared with drain removal at 5 days postoperatively. Therefore, intraoperative placement of the drain and its removal at 3 days postoperatively contributes to the prevention of postoperative SSI.

**5. Sterile dressing**

Two types of dressings are commonly used in clinical practice: sterile cotton pads, which are routinely used, and silver-containing dressings. Whether silver-containing dressings can reduce the incidence of SSI, Epstein et al. compared silver-containing dressings with conventional dressings in 234 patients undergoing OLIF and found that the use of silver-containing dressings significantly reduced the incidence of postoperative SSI compared with standard dressings.

**6. Educate patients on incision-related knowledge**

**6.1 Hospital stay after operation**

(a) Specific nursing care to achieve consistency of expertise and care.

(b) Care in accordance with post-operative care standards

(c) Nurses should inform patients of post-discharge precautions

**6.2 After discharge at home**

Note: Our center will transfer patients to local outpatient clinics or send them home before the stitches are removed at the incision site, whereas domestic hospitals often wait until the stitches are removed at the incision site before informing patients of their discharge, so we can inform them during their hospital stay.

(a) Informing patients of the need to return to the hospital

(b) Hand hygiene is required when touching the wound area and changing dressings

(c) Notify the doctor if the dressing is loose or contaminated in the surgical area

(d) Measures to keep the incision area clean.

(1) Personal hygiene: daily wiping with chlorhexidine is possible

(2) Timely diaper changes and cleanliness of the lower back

(3) Avoid hair touching the incision

(4) Use clean sheets and clothes and keep pets away from the patient's resting place

(5) No swimming (no water in the wound area) until the doctor gives permission

(6) Inform the patient of the signs and symptoms of incisional infection and give the patient the doctor's telephone number

(7) Re-examination one week after surgery
